# Systematic Affinity Purification Coupled to Mass Spectrometry Identified p62 as Part of the Cannabinoid Receptor CB2 Interactome

**DOI:** 10.3389/fnmol.2019.00224

**Published:** 2019-09-20

**Authors:** Ahmed Sharaf, Leonore Mensching, Christina Keller, Sebastian Rading, Marina Scheffold, Lysann Palkowitsch, Nevena Djogo, Meriem Rezgaoui, Hans A. Kestler, Barbara Moepps, Antonio Virgilio Failla, Meliha Karsak

**Affiliations:** ^1^Neuronal and Cellular Signal Transduction, Center for Molecular Neurobiology, University Medical Center Hamburg-Eppendorf, Hamburg, Germany; ^2^Institute of Pharmacology and Toxicology, Ulm University, Ulm, Germany; ^3^Institute of Physiological Chemistry, Ulm University, Ulm, Germany; ^4^Institute for Biochemistry and Molecular Biology, University of Hamburg, Hamburg, Germany; ^5^Institute of Medical Systems Biology, Ulm University, Ulm, Germany; ^6^Microscopy Imaging Facility, University Medical Center Hamburg-Eppendorf, Hamburg, Germany

**Keywords:** AP-MS, interactome, SQSTM1, protein–protein interaction, GPCR, endocannabinoid system

## Abstract

The endocannabinoid system (ECS) consists particularly of cannabinoid receptors 1 and 2 (CB1 and CB2), their endogenous ligands, and enzymes that synthesize and degrade their ligands. It acts in a variety of organs and disease states ranging from cancer progression over neuropathic pain to neurodegeneration. Protein components engaged in the signaling, trafficking, and homeostasis machinery of the G-protein coupled CB2, are however largely unknown. It is therefore important to identify further interaction partners to better understand CB2 receptor functions in physiology and pathophysiology. For this purpose, we used an affinity purification and mass spectrometry-based proteomics approach of Strep-HA-CB2 receptor in HEK293 cells. After subtraction of background interactions and protein frequency library assessment we could identify 83 proteins that were classified by the identification of minimally 2 unique peptides as highly probable interactors. A functional protein association network analysis obtained an interaction network with a significant enrichment of proteins functionally involved in protein metabolic process, in endoplasmic reticulum, response to stress but also in lipid metabolism and membrane organization. The network especially contains proteins involved in biosynthesis and trafficking like calnexin, Sec61A, tubulin chains TUBA1C and TUBB2B, TMED2, and TMED10. Six proteins that were only expressed in stable CB2 expressing cells were DHC24, DHRS7, GGT7, HECD3, KIAA2013, and PLS1. To exemplify the validity of our approach, we chose a candidate having a relatively low number of edges in the network to increase the likelihood of a direct protein interaction with CB2 and focused on the scaffold/phagosomal protein p62/SQSTM1. Indeed, we independently confirmed the interaction by co-immunoprecipitation and immunocytochemical colocalization studies. 3D reconstruction of confocal images furthermore showed CB2 localization in close proximity to p62 positive vesicles at the cell membrane. In summary, we provide a comprehensive repository of the CB2 interactome in HEK293 cells identified by a systematic unbiased approach, which can be used in future experiments to decipher the signaling and trafficking complex of this cannabinoid receptor. Future studies will have to analyze the exact mechanism of the p62-CB2 interaction as well as its putative role in disease pathophysiology.

## Introduction

Cellular processes are usually dependent on network dynamics and interactions of macromolecular protein complexes. Identification of interactomes of proteins and consequently connecting molecular processes are important steps for understanding complex functions such as cell signaling and receptor trafficking. Here we focused on the identification of the interactome of the CB2, which belongs to the potentially therapeutic GPCRs. GPCRs are implicated in the pathophysiology of numerous human diseases like hypertension (Brinks and Eckhart, 2010), Parkinson’s disease (Pinna et al., 2005; Gandia et al., 2013), various cancers (Lappano and Maggiolini, 2011; Xiang et al., 2018), and infectious diseases (Heng et al., 2013), thus reflecting their immense therapeutic and clinical relevance. Due to difficulties in preserving the complex three-dimensional GPCR structure during receptor solubilization the identification of their interacting partners is demanding (Daulat et al., 2009). In this context methods of tandem AP-MS and MYTH approaches have been optimized for robustness and reproducibility to obtain high-confidence protein complex information and to systematically find interactions of full-length integral membrane receptors (Daulat et al., 2009; Glatter et al., 2009; Sokolina et al., 2017). These methods have indeed been successfully used for studies on clinically relevant GPCRs such as serotonin 5-HT4d, adenosine ADORA2A receptors (Sokolina et al., 2017), and CB1 (Mattheus et al., 2016) but not for CB2.

The CB2 receptor has been shown to contribute to various pathological states in animal models especially in neurodegenerative, immunological, inflammatory, cardiovascular, hepatic, and bone disorders (Pertwee, 2012). CB2 acts as a modulator of immune suppression, induction of apoptosis, and cell migration (Basu and Dittel, 2011). In pathophysiological conditions CB2 is often upregulated in immune cells, where it inhibits proinflammatory cytokine production (Steffens et al., 2005). The pharmacological modulation of the transcription factor PPARalpha represents an important mechanism through which CB2 expression could be regulated (Guida et al., 2017). Of note, CB2 receptors are involved in the regulation of co-stimulatory factors, such as LPS and TNF-alpha, which signal via distinct membrane receptors (Toguri et al., 2014). Hence, it is of interest to identify interactors of CB2 that might act as signaling hubs for the crosstalk of different signaling events or that regulate the trafficking and degradation of the receptor.

By an AP-MS approach we have now identified p62 (also named sequestosome 1, SQSTM1) as an interacting partner of CB2 receptor. The scaffold protein p62 contains various domains that modulate protein–protein interactions; p62, e.g., associates with TRAF6 and with the death-domain kinase RIP1 and regulates NF-κB (nuclear factor- light-chain-enhancer of activated B cells) and MAP kinase signaling downstream of the TNF receptor, RANK receptor and nerve growth factor receptor (Moscat et al., 2007). Mutations in p62 are associated with neurodegenerative diseases and with PDB. The latter is characterized by increased bone remodeling in focal areas leading to bone pain, deformities and fractures (Laurin et al., 2002; Seitz et al., 2008). A P392L exchange found in the UBA of the C-terminal part of p62 protein is a prominent mutation present in patients with PDB and with amyotrophic lateral sclerosis and/or frontotemporal dementia (Laurin et al., 2002; Cavey et al., 2006; Moscat et al., 2007; Fecto et al., 2011). This UBA domain is also of importance for the interaction of p62 with polyubiquitinated proteins, destined for degradation by autophagy (Moscat and Diaz-Meco, 2011). Through its interaction with LC3B p62 recruits ubiquitinated cargo proteins to the autophagosomal degradation pathway (Pankiv et al., 2007). Defects in autophagy lead to an accumulation of p62 (Komatsu et al., 2007). P62 aggregates are highly accumulated in neurons and glial cells in patients with neurodegenerative diseases (Seilhean et al., 2009; Al-Sarraj et al., 2011). Interestingly, in a number of neurodegenerative disorders a CB2 upregulation especially in microglial cells has been described and neuroprotective effects of CB2 ligands were shown (Cassano et al., 2017).

In summary, with a systematic unbiased approach we identified the CB2 interactome. Our data will serve in future studies to decipher cannabinoid receptor trafficking and signaling complexes and receptor homeostasis. We could furthermore validate our approach by confirming an interaction of CB2 with p62. Interestingly, it has been shown that the dysfunction of both proteins alone is critically involved in related disorders affecting brain and bone (Pacher and Mechoulam, 2011; Duran et al., 2016; Sanchez-Martin and Komatsu, 2018), so that future studies will have to analyze the exact mechanism of this interaction and investigate its role in pathophysiological conditions.

## Materials and Methods

### Antibodies and Reagents

The following antibodies and chemicals were used: Rabbit anti-p62 (#P0067, Sigma), mouse anti-CB2 (#H00001269-M01, Abnova), secondary antibodies; Alexa Fluor^®^488 goat anti-mouse (#A21202, Thermo Fisher Scientific), Alexa Fluor^®^649 goat anti-rabbit (#111-496-144, JacksonImmuno Research), rabbit anti-FLAG (#F7425, Sigma). Lectin WGA conjugated with Texas Red^®^-X (#W7024, Thermo Fisher Scientific) HU-210 from Tocris (CAS No. 112830-95-2), poly-L-Lysine (#P6282-5mg, Sigma), bovine serum albumin (#A9418, Sigma), Triton X-100 (#A4975, AppliChem), ProLong^®^ Gold antifade reagent with DAPI (#P36935, Thermo Fisher Scientific), HBSS (#H6648, Sigma).

### Plasmids and Cell Culture

Human HEK293 cells (obtained from ATCC) were cultured in Dulbecco’s modified Eagle’s medium (Thermo Fisher Scientific) supplemented with 10% FBS, penicillin (50 units/ml), and streptomycin (50 μg/ml) at 37°C, 5% CO_2_. Transient transfections were performed with Lipofectamine 2000 (Thermo Fisher Scientific) according to the manufacturer’s protocol.

Flag-p62 and CB2 expression vectors were generated by inserting human cDNA in-frame into EcoRI and XhoI sites into the pcDNA3.1 vector (Thermo Fisher Scientific). The deletion constructs for p62 and CB2 were generated by site-directed mutagenesis using the QuikChange site directed mutagenesis kit (#200523, Agilent) according to the manufacturer’s protocol. The ΔC-CB2 construct was generated by PCR amplification introducing a stop codon after the amino acid position 300 of human CB2 protein sequence. CB2 deletion constructs of intracellular loops (Δi) and p62 deletion constructs via XL II Gold Site-Directed Mutagenesis Kit (#200521, Agilent) were generated using following primer pairs (Table 1).

**TABLE 1:** Sequences of primer pairs used for the generation of CB2 deletion constructs of intracellular loops (Δi) and p62 deletion constructs.

**Flag-plasmids**	**Forward primer (5′–3′)**	**Reverse primer (5′–3′)**
Δi1-CB2	GTGCTCTATCTGATCCTGCTGTTCATTGGCAGCTTG	CAAGCTGCCAATGAACAGCAGGATCAGATAGAGCAC
Δi2-CB2	CCTCCTGCTGACCGCCATTGCACTGGTGA	TCACCAGTGCAATGGCGGTCAGCAGGAGG
Δi3-CB2	GGGCATGTTCTCTGGACCCTAGGGCTAGTG	CACTAGCCCTAGGGTCCAGAGAACATGCCC
p62-ΔZZ122-167	AGGGGCTGGGGAACATGTTGCGGGGC	GCCCCGCAACATGTTCCCCAGCCCCT
P62-ΔPEST266-294	CCCACCCGGCTTTCTTTTCCCTCCGTGCT	AGCACGGAGGGAAAAGAAAGCCGGGTGGG
P62-ΔUBA389-434	CACATCTCCCGCCAAAGCATCCCCCGCC	GGCGGGGGATGCTTTGGCGGGAGATGTG

### Bait Cloning and Stable Cell Line (Dualsystems Biotech AG)

The human open reading frame of the CB2 receptor (NM_001841.3; corresponding to NP_001832) was inserted into the bait vector pN-TGSH (Dualsystems). Bait expression construct pN-TGSH/CB2 with N-terminal Strep-HA fusion was used for the generation of a stable cell line using the FRT-HEK293 cells (Thermo Fisher Scientific) containing a single genomic FRT site supporting the rapid generation of bait-expressing cell lines by Flp-mediated recombination (O’Gorman et al., 1991; Glatter et al., 2009). Stable cell line HEK293/CB2 was cultured in complete medium (D-MEM (high glucose), 10% FBS, 2 mM L-glutamine, 100 ug/ml Hygromycin) at 37°C, 5% CO_2_.

### Affinity Purification and Mass Spectrometric Analysis

The Dualsystems CaptiVate approach including LC–MS/MS analysis and peptide assignment were described previously in detail (Glatter et al., 2009). For the analysis of stable cell line and bait expression protein extraction was performed with 0.5% DDM containing affinity purification lysis buffer (Dualsystems) followed by a 12% SDS-PAGE and Western blotting. A mouse monoclonal anti-HA antibody was used for bait detection. Calculated mass of the fusion protein was 50 kDa. Pulldown was performed with CB2 expressing and non-expressing control Flp-In HEK293 cell lysates and 100 μl Strep-Tactin Sepharose. Affinity purification and mass spectrometric analysis of Strep-HA CB2 was performed in three biological replicates for quantitative mass spectrometric analysis and monitored by Western blotting. The samples were analyzed on a Thermo LTQ Orbitrap XL spectrometer using a C18 column, ESI and a 60 min gradient. The variation of the biological replicates of untransfected (replicates 1–3 control cells) or Strep-HA CB2 expressing HEK293 cells (replicates 1–3 CB2 cells) was below 10% that was calculated as Pearson’s correlation coefficient of variation (r) by GraphPad Prism software. All steps of the CaptiVate approach were done by Dualsystems Biotech AG (Schlieren, Switzerland) and were described in details previously (Glatter et al., 2009). Results and experimental parameters for mass spectrometric analysis and protein identification are given in the Supplementary Tables 1–6.

### Background Subtraction

To define the background the affinity purified sample without CB2 (control sample) was used as a negative control experiment. Such a negative control should reproduce a maximum of unspecific bindings that can be subtracted to identify the interacting portion of the obtained peptides. Therefore, we calculated log2 of intensity of probe samples versus control samples (column B) with the range of >1,000 and analyzed the normal distribution (Gauss curve) by counting and distributing them on a bin histogram. For the whole range of log2 values defined bins with increments of 0.2 were set. The adjusted values were multiplicated by total number of values in column B (used for frequency distribution calculation). The threshold was set to 10% of the maximal peak height at 5.3152.

### Protein Interaction Analysis

All identified proteins that were detected by more than one unique peptide were used for the generation of an interaction network using the STRING program on https://string-db.org/ (Szklarczyk et al., 2015). The top five results of GO analysis categorized into BP, MF, and CC were also obtained by STRING database. Further GO annotations were performed by the DAVID Bioinformatics Database^1^ (Huang da et al., 2009a, b). The enrichment score represents the overall enrichment for a group based on the EASE Score of each term members. EASE Score is a modified Fisher Exact *p*-Value, for gene-enrichment analysis. GraphPad PRISM software (Version 7) was used for linear regression and Gaussian distribution.

### Immunocytochemistry

HEK293 cells were plated on poly-L-Lysine coated glass cover slips and transfected with CB2-plasmid (2 μg) with Lipofectamine 2000. After 24 h of transfection cells were washed with HBSS and fixed with Rotifix (4% PFA/PBS) for 15 min at 37°C. After washing the cells were stained with the lectin WGA conjugated with texas red (Texas Red^®^-X conjugate of WGA 1:200, 15 min, RT) which binds to sialic acid and *N*-acetylglucosaminyl residues that is used as a marker for plasma membranes before permeabilization. After three washing steps cells were permeabilized with 2% Triton X-100 in 2% BSA in PBS for 45 min at RT on a shaker followed by an incubation with primary antibodies for CB2 (Abnova, 1:300) and p62 (Sigma, 1:300) overnight at 4°C in 2% BSA-PBS. Secondary antibodies (1:1,000 in 2% BSA and 1% normal horse serum in PBS) were incubated for 1 h at RT, cells were washed and mounted with ProLong^®^ Gold antifade reagent with DAPI.

### Imaging and 3D Reconstruction Modeling

Four colors images were acquired by mean of a confocal laser scan microscope, i.e., Leica TCS SP5. As excitation four laser lines, 405, 488, 561, 633 nm were employed. For excitation and for detection of the fluorescence signal a 63x NA 1.4 oil objective lens was used. An AOBS separated the detected light from the excitation signal. The fluorescence signal, after being spectrally separated, was directed to a HyD. An estimation of the p62 co-localization with CB2 was performed by using the Manders coefficient. Therefore, we imaged a field of view including approximately 10 cells from eight coverslips in an area of approximately 125 μm^2^ with a pixel size of approximately 81 nm and a z-stack spacing of 300 nm. The laser light and detector gain were set in a way that the saturation of the detected signal was avoided. After repetitive measurements the background was estimated to be equal to 10/5% of the peak signal for p62/CB2 channels respectively and was subtracted in each channel accordingly. All the post-processing analysis and evaluation were performed with the software Bitplane Imaris. The mean values obtained per image were used for the calculation of mean ± SD. In all the three-dimensional acquisitions the lateral (xy) pixel size was set to be 100 nm (x, y) and the transversal pixel size was set to be 300 nm, i.e., the distance between image planes within a stack (z). Laser intensities and detection gain were to be the same in order to perform comparative studies between the acquired images. The software Bitplane Imaris was used to visualize and study the confocal pictures. As preliminary data visualization the signal offset was determined and subtracted from each image channel. Afterward, a segmentation algorithm was used for visualizing the spatial distribution of the samples under investigation. This algorithm uses a variable intensity threshold based on the local variation of signal to offset ratio to isolate a structure and it was the ideal choice for analyzing objects that, like the ones under investigation, are not continuous within the image volume. Finally, all signal above the local threshold belongs to the structure and all signal under the threshold are set to zero. For each fluorophore a threshold value was determined that represented an optimal coverage of the raw images. Segmented images were than displayed and image snapshots were taken at different visualization angles as well as different zoom factors.

### Stimulation of HEK293 Cells With CB2- Synthetic Cannabinoids

Transiently transfected HEK293 cells were stimulated with 100 nM HU-210 for 10 min about 24 h after transfection. Therefore, medium was aspirated and 500 μl of DMEM containing 10% FCS and synthetic cannabinoid or solvent control (ethanol), respectively, were added. HEK293 cells were incubated in 5% CO_2_ atmosphere at 37°C. Afterward, cell lysates were prepared as described in the following paragraphs.

### Co-immunoprecipitation

To immunoprecipitate Flag-tagged proteins transiently transfected HEK293 cells in 6-well cell culture plate were used. About 24 h after transfection the medium of the HEK293 cells was aspirated and the cells were washed with 800 μl ice-cold PBS from the plate and transferred to a 1.5 ml reaction vessel. After a centrifugation step at 8.000 × *g* at RT for 2 min supernatant was removed and the cell pellet was resuspended in 120 μl 0.2% DDM lysis buffer. Cell suspension was incubated by end-over-end rotation at 4°C for 30 min and centrifuged at 16.200 × *g* at 4°C for 15 min to remove cell debris. Cell lysates could be directly used for immunoprecipitation or stored at −80°C. 20 μl of cell lysates were used as input control, to this end it was mixed with 5 μl SDS loading buffer and stored on ice until SDS-PAGE. For each immunoprecipitation sample 8 μl anti-Flag^®^ M2 affinity gel was prepared. For that purpose, the whole volume of anti-Flag^®^ M2 affinity gel was washed three times with about 500 μl 0.2% DDM lysis buffer. For each sample 100 μl 0.2% DDM lysis buffer was added to the washed anti-Flag^®^ M2 affinity gel. The remaining cell lysate was diluted with 200 μl 0.2% DDM lysis buffer and 100 μl anti-Flag^®^ M2 affinity gel were added and incubated by end-over-end rotation at 4°C for 1 h. The immunoprecipitates were washed three times with 500 μl lysis buffer containing 0.2% DDM and one time with 500 μl ice-cold PBS. Remaining supernatant was removed with a syringe and cannula. After washing 18 μl SDS loading buffer were added to the immunoprecipitates which were incubated at 70°C for 10 min. The input samples were not heated. Immunoprecipitates and input control samples were analyzed by SDS-PAGE and Western blotting.

### Western Blotting Analysis

HEK293 cell lysates were mixed with 6x Laemmli buffer [100 mM Tris-HCl (pH 6.8), 4% SDS, 60% glycerol, 0.2% bromophenol blue, and 10 mM DTT in dH_2_O]. Proteins of the cell lysate were separated via SDS-PAGE with 12% gels and transferred to Amersham Hybond ECL nitrocellulose membrane (#RPN2032D, GE Healthcare, United Kingdom). After blocking with 5% skimmed milk powder in TBS-T (150 mM NaCl, 10 mM Tris and 0.025% Tween^®^ 20 in dH_2_O), the membrane was incubated with specific primary antibodies in TBS-T or blocking buffer over night at 4°C. Afterward, fluorescence dye-conjugated or HRP-coupled secondary antibodies were incubated for 1 h at RT. After washing the proteins were detected with the two-channel IR direct detection imaging system from Odyssey LI-COR, United States, or by chemiluminescence. Signal quantifications were done using the Image Studio Software 5.2.5 of LI-COR Bioscience.

## Results

### Analysis of Affinity Purified CB2 Receptor Complexes

**FIGURE 1:**
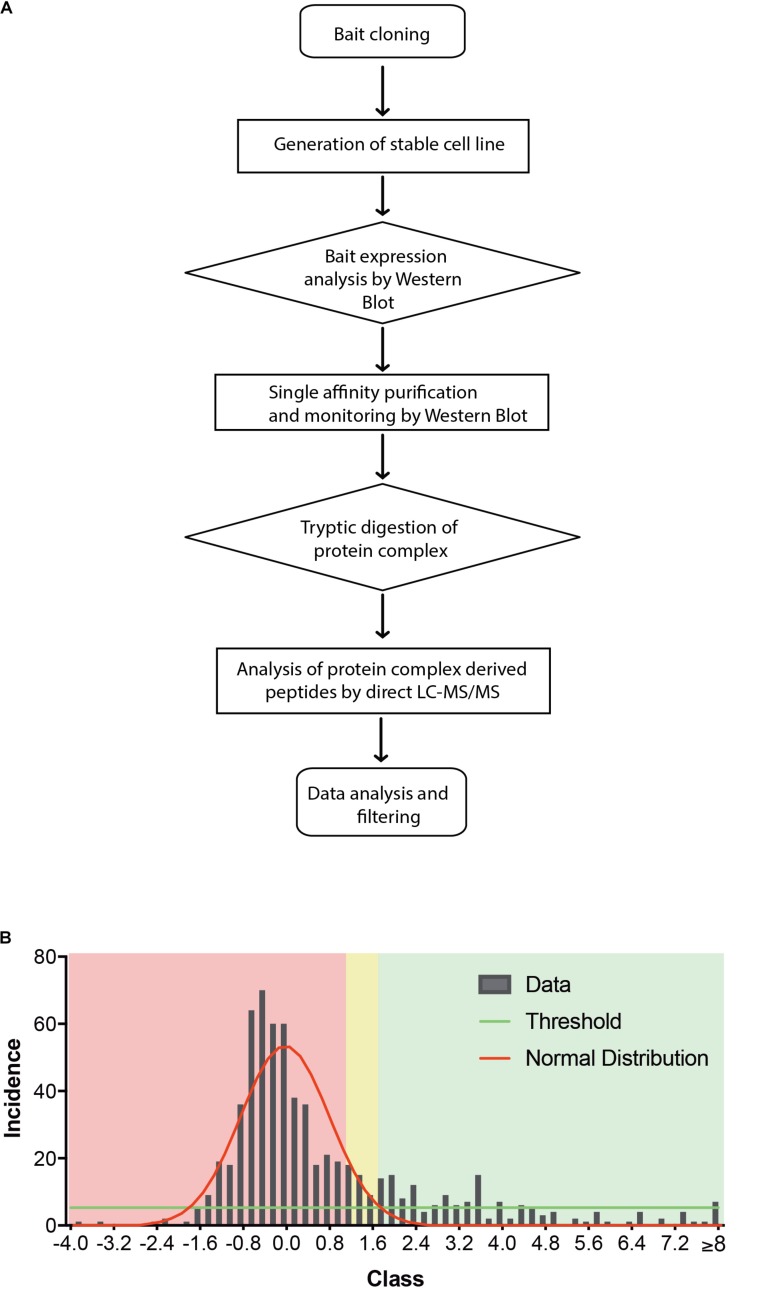
**(A)** Flow chart of CaptiVate screening procedure. The bait CB2 was amplified and sub-cloned into the expression vector pN-TGSH (Dualsystems). The bait sequence was verified by sequencing. For constitutive expression, the bait was transfected into HEK293 Flp-In cells and selected for stable integration. Bait expression and solubility was monitored by immunoblotting using anti-HA antibodies. The expression level was compared to an internal standard and judged suitable for affinity purification and mass spectrometric analysis. **(B)** Background filtering of CB2 affinity purification: histogram of enrichment factors (CB2/control) of identifications to determine threshold of enrichment for specific interactors. Red area represents Gaussian distribution of unspecific interactors, yellow area is in the border and in the green area are the specific interactors (threshold as 10% of maximum peak height).

Most biochemical investigations of GPCRs including CB2 are methodologically challenging due to their topology as multiple transmembrane proteins, that is vulnerable to common extraction procedures. We therefore used a method especially applicable for membrane receptors combining systematic affinity purification and mass spectrometric analysis to identify CB2 putative interaction partners (Figure 1A, CaptiVate Dualsystems, performed by Dualsystems Biotech AG, Switzerland) (Glatter et al., 2009). Due to the lack of specific and reliable antibodies for a detection of endogenous CB2 receptors (Cecyre et al., 2014), we were restricted in our studies to use heterologous expression systems. To this aim the bait CB2 was sub-cloned and stably expressed in HEK293 Flp-In cells. Affinity purification and quantitative mass spectrometric analysis (LC–MS/MS) of Strep-HA CB2 were performed in three biological replicates. The samples analyzed showed a variation below 10% of the biological replicates of untransfected or Strep-HA CB2 expressing HEK293 cells (Supplementary Figure 1).

Indeed, a total of 39.2% of the CB2 protein sequence was covered by mass spectrometric data. In total 698 proteins were identified. As expected for a GPCR guanine-nucleotide-binding protein G subunits alpha GNAI3 and GNAS1 have each been identified by one unique peptide. After subtraction of background interactions (Figure 1B) and protein frequency library assessment (Glatter et al., 2009) we could identify 83 putative interactors that were classified by the identification of minimally two unique peptides as highly probable interactors (Table 2).

**TABLE 2:** CB2 interactors in HEK293 cells.

**Entry**	**Entry name**	**Protein names**	**Unique**	**Sequence**	**Molecular**	**Ratio**
			**peptides**	**coverage [%]**	**weight [kDa]**	**CB2/ctrl**
P34972	CNR2_HUMAN	Cannabinoid receptor 2	16	39.2	39.68	845.0
Q15392	DHC24_HUMAN	Delta(24)-sterol reductase	2	5.4	60.101	Only in CB2
Q9Y394	DHRS7_HUMAN	Dehydrogenase/reductase SDR family member 7	2	14.2	38.298	Only in CB2
Q9UJ14	GGT7_HUMAN	Glutathione hydrolase 7	4	11.2	70.466	Only in CB2
Q5T447	HECD3_HUMAN	E3 ubiquitin-protein ligase HECTD3	2	3.8	97.112	Only in CB2
Q8IYS2	K2013_HUMAN	Uncharacterized protein KIAA2013	4	11.1	72.68	Only in CB2
O15162	PLS1_HUMAN	Phospholipid scramblase 1	4	14.8	35.049	Only in CB2
O14967	CLGN_HUMAN	Calmegin	19	45.7	70.038	12388.6
Q06136	KDSR_HUMAN	3-Ketodihydrosphingosine reductase (KDS reductase)	7	32.2	36.187	2214.8
Q96JJ7	TMX3_HUMAN	Protein disulfide-isomerase TMX3	13	39	51.871	551.6
Q99720	SGMR1_HUMAN	Sigma non-opioid intracellular receptor 1	6	40.4	25.127	370.9
P16615	AT2A2_HUMAN	Sarcoplasmic/endoplasmic reticulum calcium ATPase 2 (SERCA2)	44	48.6	114.76	306.2
P27824	CALX_HUMAN	Calnexin (IP90) (Major histocompatibility complex class I antigen-binding protein p88)	38	57.9	67.567	200.4
Q8IWV7	UBR1_HUMAN	E3 ubiquitin-protein ligase UBR1	3	2.8	200.21	170.0
P37268	FDFT_HUMAN	Squalene synthase	8	24.5	48.115	168.0
Q99442	SEC62_HUMAN	Translocation protein SEC62	4	10	45.861	164.7
O43505	B4GA1_HUMAN	Beta-1.4-glucuronyltransferase 1	2	12.8	47.119	161.7
Q9UBV2	SE1L1_HUMAN	Protein sel-1 homolog 1	2	6.2	88.754	124.8
P10586	PTPRF_HUMAN	Receptor-type tyrosine-protein phosphatase F	5	4.5	212.88	88.1
O95260	ATE1_HUMAN	Arginyl-tRNA–protein transferase 1	4	10.2	59.09	87.4
Q96A33	CCD47_HUMAN	Coiled-coil domain-containing protein 47	11	34.4	55.873	85.3
O15269	SPTC1_HUMAN	Serine palmitoyltransferase 1	4	11.8	52.743	77.3
Q4ZIN3	MBRL_HUMAN	Membralin (Transmembrane protein 259)	3	7.3	67.888	62.9
O43149	ZZEF1_HUMAN	Zinc finger ZZ-type and EF-hand domain-containing protein 1	5	2.8	331.07	55.5
Q13501	SQSTM_HUMAN	Sequestosome-1	6	23.4	47.687	52.7
O15258	RER1_HUMAN	Protein RER1	2	15.3	22.958	51.5
Q9H3N1	TMX1_HUMAN	Thioredoxin-related transmembrane protein 1	3	12.9	31.791	42.2
O00264	PGRC1_HUMAN	Membrane-associated progesterone receptor component 1	8	57.4	21.671	40.5
Q15043	S39AE_HUMAN	Zinc transporter ZIP14 (LIV-1 subfamily of ZIP zinc transporter 4)	2	6.7	54.212	31.2
Q8NI60	COQ8A_HUMAN	Atypical kinase COQ8A mitochondrial	3	8.3	71.949	28.5
P55084	ECHB_HUMAN	Trifunctional enzyme subunit beta mitochondrial	15	43.7	51.294	27.8
P42356	PI4KA_HUMAN	Phosphatidylinositol 4-kinase alpha	2	1.6	231.32	26.3
O75915	PRAF3_HUMAN	PRA1 family protein 3 (ADP-ribosylation factor-like protein 6-interacting protein 5)	2	16	21.614	25.6
A1L0T0	ILVBL_HUMAN	Acetolactate synthase-like protein	4	10.9	67.867	23.1
Q9Y4P3	TBL2_HUMAN	Transducin beta-like protein 2	2	5.6	49.797	22.5
O60884	DNJA2_HUMAN	DnaJ homolog subfamily A member 2	2	6.8	45.745	22.2
Q29963	1C06_HUMAN	HLA class I histocompatibility antigen. Cw-6 alpha chain (MHC class I antigen Cw^∗^6)	5	21.9	40.968	21.5
P20020	AT2B1_HUMAN	Plasma membrane calcium-transporting ATPase 1	3	4.2	138.75	21.1
P49755	TMEDA_HUMAN	Transmembrane emp24 domain-containing protein 10	2	12.8	24.976	19.8
P62341	SELT_HUMAN	Thioredoxin reductase-like selenoprotein T (SelT)	2	17.4	22.174	18.8
P50395	GDIB_HUMAN	Rab GDP dissociation inhibitor beta	2	8.5	50.663	17.0
P49411	EFTU_HUMAN	Elongation factor Tu mitochondrial (EF-Tu)	23	61.5	49.541	15.2
Q8IXI1	MIRO2_HUMAN	Mitochondrial Rho GTPase 2 (MIRO-2) (hMiro-2)	2	4.4	68.117	15.2
Q14257	RCN2_HUMAN	Reticulocalbin-2	2	11	36.876	14.6
Q9BQE3	TBA1C_HUMAN	Tubulin alpha-1C chain (Alpha-tubulin 6)	2	76.2	49.895	12.4
Q9BXW9	FACD2_HUMAN	Fanconi anemia group D2 protein (Protein FACD2)	2	2.3	166.46	12.1
P04844	RPN2_HUMAN	Dolichyl-diphosphooligosaccharide–protein glycosyltransferase subunit 2	11	37.9	69.283	12.1
P04843	RPN1_HUMAN	Dolichyl-diphosphooligosaccharide–protein glycosyltransferase subunit 1	19	43	68.569	12.0
P40939	ECHA_HUMAN	Trifunctional enzyme subunit alpha mitochondrial	27	53.1	82.999	12.0
P39656	OST48_HUMAN	Dolichyl-diphosphooligosaccharide–protein glycosyltransferase 48 kDa subunit	11	45.2	50.8	11.9
Q9BVA1	TBB2B_HUMAN	Tubulin beta-2B chain	3	67.2	49.953	11.5
Q9BQB6	VKOR1_HUMAN	Vitamin K epoxide reductase complex subunit 1	2	19	18.234	11.1
P13797	PLST_HUMAN	Plastin-3 (T-plastin)	2	6.3	70.81	11.0
Q9HCU5	PREB_HUMAN	Prolactin regulatory element-binding protein	3	15.1	45.468	10.8
P07237	PDIA1_HUMAN	Protein disulfide-isomerase (PDI)	2	4.3	57.116	10.3
P51648	AL3A2_HUMAN	Fatty aldehyde dehydrogenase	6	14.4	57.669	9.9
P13073	COX41_HUMAN	Cytochrome c oxidase subunit 4 isoform 1 mitochondrial	2	13.6	19.576	9.0
P08195	4F2_HUMAN	4F2 cell-surface antigen heavy chain (4F2hc)	3	7	71.122	9.0
Q15363	TMED2_HUMAN	Transmembrane emp24 domain-containing protein 2 (Membrane protein p24A)	2	10.9	22.761	8.8
P61204	ARF3_HUMAN	ADP-ribosylation factor 3	2	18.2	20.601	8.8
O15173	PGRC2_HUMAN	Membrane-associated progesterone receptor component 2	2	18.8	23.818	7.9
O95831	AIFM1_HUMAN	Apoptosis-inducing factor 1 mitochondrial	5	11.1	66.9	6.8
P00403	COX2_HUMAN	Cytochrome c oxidase subunit 2	3	16.3	25.565	6.1
P05109	S10A8_HUMAN	Protein S100-A8 (Calgranulin-A)	3	32.3	10.834	6.0
Q12931	TRAP1_HUMAN	Heat shock protein 75 kDa mitochondrial (HSP 75) (TNFR-associated protein 1)	7	13.2	80.109	5.5
P22102	PUR2_HUMAN	Trifunctional purine biosynthetic protein adenosine-3	4	7.5	107.77	5.4
O14975	S27A2_HUMAN	Very long-chain acyl-CoA synthetase (VLACS) (VLCS)	2	5.5	70.311	5.2
P11586	C1TC_HUMAN	C-1-Tetrahydrofolate synthase cytoplasmic (C1-THF synthase)	2	2.7	101.56	5.1
O75396	SC22B_HUMAN	Vesicle-trafficking protein SEC22b (ER-Golgi SNARE of 24 kDa)	2	10.2	24.593	5.1
P30101	PDIA3_HUMAN	Protein disulfide-isomerase A3	5	13.9	56.782	5.1
P53618	COPB_HUMAN	Coatomer subunit beta (Beta-coat protein)	2	2.2	107.14	4.9
P51571	SSRD_HUMAN	Translocon-associated protein subunit delta (TRAP-delta)	2	18.5	18.998	4.8
P61619	S61A1_HUMAN	Protein transport protein Sec61 subunit alpha isoform 1 (Sec61 alpha-1)	2	9	52.264	4.6
P30048	PRDX3_HUMAN	Thioredoxin-dependent peroxide reductase mitochondrial	2	9.8	27.692	4.5
Q7Z6Z7	HUWE1_HUMAN	E3 ubiquitin-protein ligase HUWE1	2	0.5	481.89	4.3
P50402	EMD_HUMAN	Emerin	3	17.3	28.994	4.3
P24534	EF1B_HUMAN	Elongation factor 1-beta (EF-1-beta)	3	26.7	24.763	4.1
Q53GQ0	DHB12_HUMAN	Very-long-chain 3-oxoacyl-CoA reductase	4	21.2	34.324	4.0
Q9P035	HACD3_HUMAN	Very-long-chain (3R)-3-hydroxyacyl-CoA dehydratase 3	9	33.1	43.159	3.8
Q00325	MPCP_HUMAN	Phosphate carrier protein mitochondrial (Phosphate transport protein) (PTP)	8	28.5	39.958	3.7
O14980	XPO1_HUMAN	Exportin-1	5	8.3	123.38	3.7
P06702	S10A9_HUMAN	Protein S100-A9 (Calgranulin-B)	3	36	13.242	3.7
Q86VU5	CMTD1_HUMAN	Catechol *O*-methyltransferase domain-containing protein 1	4	25.2	28.808	3.6
Q93008	USP9X_HUMAN	Probable ubiquitin carboxyl-terminal hydrolase FAF-X	3	1.7	292.28	3.6

*All specific interactors are classified by the identification in mass spectrometry data of triplicates by at least two unique peptides. The Uniprot accession numbers with protein name, descriptions and molecular weights are given. Sequence coverage by identified peptides is presented in %. Additionally, the ratio of identification in CB2 expressing cells to control cells was calculated.*

**FIGURE 2:**
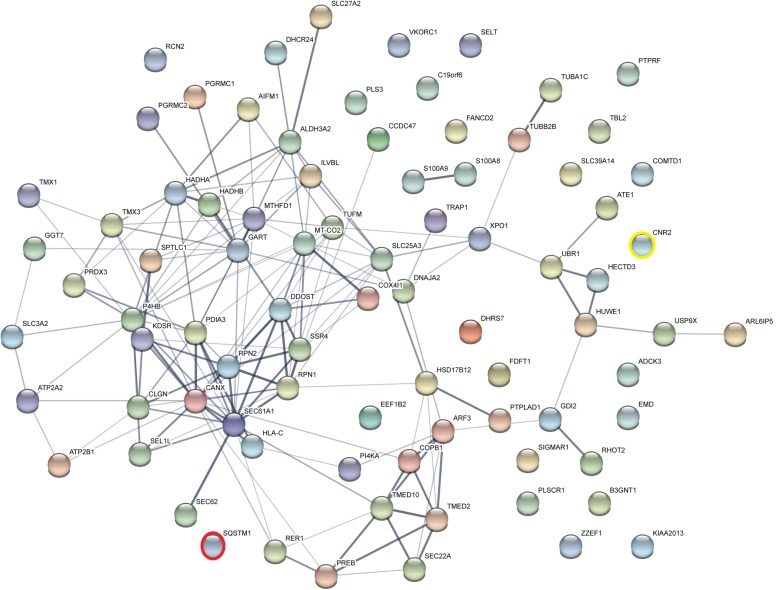
Functional Annotations were used to visualize protein–protein interaction network of 83 identified CB2 interactors by STRING. Illustrated interaction network was determined by text mining, experiments or databases with a minimum required interaction score of 0.400. In this view, the nodes represent proteins and the color saturation of the edges represents the confidence score of data support. CNR2 represents CB2 and SQSTM1 represents p62.

Taking those 83 putative interactors we processed a functional protein association network analysis using the STRING online tool (Szklarczyk et al., 2015) obtaining an interaction network with a protein–protein-interaction enrichment *p*-value < 1.0e-16. This network consists of 84 nodes (including CB2) and 163 edges with an average node degree of 3.8 and an average local clustering coefficient of 0.392. In the network a significant functional enrichment of proteins involved in protein metabolic process and in response to endoplasmic reticulum (ER) stress were obtained using the GO BP analysis (Table 3). These functions are in line with the findings of CC ontology revealing an annotation of ER in 35 CB2 interacting proteins (Table 3). Further, the MF gene ontology analysis unveiled an enrichment of proteins with catalytic activity and oxidoreductase activity (Table 3). Functional annotations that were used by the STRING database to illustrate interactions network were determined by: text mining, experiments, or databases with a minimum required interaction score of 0.400 (Figure 2). The nodes represent proteins whereas color saturation of the edges mark confidence of the interactions indicating the strength of data support. Several proteins were not connected by any known interactions to the other proteins and are displayed as nodes without any edges. In contrast, some proteins particularly located in the center of the network with many interactions are ER proteins like calnexin and Sec61A1. Also the cargo receptors for GPCR trafficking and resensitization TMED2 and TMED10 have been identified in this network.

**TABLE 3:** Gene ontology (GO) analysis of 84 CB2 specific interactors (including CB2) using the STRING database (https://string-db.org/).

**Biological process (GO)**			

**Pathway ID**	**Pathway description**	**Count in**	**False discovery**
		**gene set**	**rate**
GO:0019538	Protein metabolic process	37	2.8e-05
GO:0034976	Response to endoplasmic reticulum stress	10	2.8e-05
GO:0044267	Cellular protein metabolic process	34	2.8e-05
GO:0006457	Protein folding	9	0.000337
GO:0061024	Membrane organization	16	0.000342
**Molecular function (GO)**			
GO:0003824	Catalytic activity	41	0.000457
GO:0016491	Oxidoreductase activity	14	0.000457
GO:0003756	Protein disulfide isomerase activity	4	0.00114
GO:0016408	C-Acyltransferase activity	3	0.0118
GO:0016509	Long-chain-3-hydroxyacyl-CoA dehydrogenase activity	2	0.0118
**Cellular component (GO)**			
GO:0042175	Nuclear outer membrane-endoplasmic reticulum membrane network	31	3.40e-17
GO:0044432	Endoplasmic reticulum part	29	1.17e-16
GO:0005789	Endoplasmic reticulum membrane	31	1.17e-16
GO:0005783	Endoplasmic reticulum	35	4.01e-16
GO:0031090	Organelle membrane	42	4.50e-13

*Displayed are the most significant top five GO pathways in the category Biological Process (BP), Molecular Function (MF), Cellular Component (CC).*

To identify further functional annotations, a systematic analysis of all 83 CB2 interactors was performed using the “DAVID Functional Annotation Tool” (Huang da et al., 2009a, b). The analysis identified 20 clusters with 10 clusters having an enrichment score higher than 2.0 (Table 4). The top five of these clusters with a highly significant enrichment (*p* < 0.0001) in the obtained CB2 interactome (Table 4) represent the ER (cluster 1), membrane (cluster 2), oxidoreductase (cluster 3), ER stress and cell redox homeostasis (cluster 4), and lipid metabolism (cluster 5). As the CB2 receptor is expected to interact with nucleotide binding G protein subunits, we screened the functional annotation list with the keyword “nucleotide binding” and identified 13 proteins belonging to this cluster (*p*-value = 5.4 e-2). Out of them five CB2 interactors were enriched for the GOTerm “GTP binding,” namely the ADP-ribosylation factor 3 (ARF3), the translation elongation factor TUFM, the member of the Rho family of GTPases RHOT2, and the tubulin chains TUBA1C and TUBB2B (*p*-value of 2.5 e-2).

**TABLE 4:** Gene ontology (GO) functional annotation clustering.

		**Count**	***p*_Value**	**FDR**
**Cluster 1**	**Enrichment score: 15.5**			
UP_KEYWORDS	Endoplasmic reticulum	34	2.7E-21	3.3E-18
GOTERM_CC_DIRECT	Endoplasmic reticulum membrane	27	3.2E-15	3.8E-12
UP_SEQ_FEATURE	Topological domain:lumenal	18	3.6E-12	4.9E-9
**Cluster 2**	**Enrichment score: 9.61**			
UP_KEYWORDS	Membrane	63	1.3E-12	1.6E-9
UP_KEYWORDS	Transmembrane helix	54	4.0E-12	5.0E-9
UP_KEYWORDS	Transmembrane	54	4.6E-12	5.6E-9
UP_SEQ_FEATURE	Transmembrane region	48	1.1E-9	1.5E-6
GOTERM_CC_DIRECT	Integral component of membrane	50	2.7E-9	3.2E-6
UP_SEQ_FEATURE	Topological domain:cytoplasmic	33	3.1E-6	4.3E-3
**Cluster 3**	**Enrichment score: 3.59**			
UP_KEYWORDS	Oxidoreductase	12	2.1E-5	2.6E-2
GOTERM_BP_DIRECT	Oxidation–reduction process	12	7.7E-5	1.1E-1
GOTERM_MF_DIRECT	Oxidoreductase activity	7	3.7E-4	4.7E-1
UP_KEYWORDS	NADP	5	6.8E-3	8.1E0
**Cluster 4**	**Enrichment score: 3.17**			
UP_KEYWORDS	Redox-active center	7	4.4E-8	5.4E-5
GOTERM_BP_DIRECT	Response to endoplasmic reticulum stress	6	2.4E-5	3.4E-2
GOTERM_BP_DIRECT	Cell redox homeostasis	6	2.7E-5	3.9E-2
INTERPRO	Thioredoxin domain	5	3.0E-5	3.9E-2
INTERPRO	Thioredoxin, conserved site	4	5.2E-5	6.8E-2
GOTERM_MF_DIRECT	Protein disulfide isomerase activity	4	1.5E-4	1.9E-1
INTERPRO	Thioredoxin-like fold	6	2.6E-4	3.4E-1
GOTERM_CC_DIRECT	Cell	5	1.1E-3	1.3E0
UP_SEQ_FEATURE	Short sequence motif:prevents secretion from ER	4	2.1E-3	2.8E0
GOTERM_MF_DIRECT	Isomerase activity	3	4.4E-3	5.4E0
UP_SEQ_FEATURE	Domain:thioredoxin	3	8.2E-3	1.1E1
UP_KEYWORDS	Isomerase	3	8.7E-2	6.7E1
UP_SEQ_FEATURE	Disulfide bond	14	4.3E-1	1.0E2
UP_KEYWORDS	Disulfide bond	13	7.5E-1	1.0E2
**Cluster 5**	**Enrichment score: 2.87**			
GOTERM_MF_DIRECT	Long-chain-3-hydroxyacyl-CoA dehydrogenase activity	3	6.6E-5	8.3E-2
UP_KEYWORDS	Lipid metabolism	9	3.6E-4	4.4E-1
KEGG_PATHWAY	Fatty acid elongation	4	5.4E-4	5.8E-1
KEGG_PATHWAY	Fatty acid metabolism	4	3.6E-3	3.8E0
KEGG_PATHWAY	Biosynthesis of unsaturated fatty acids	3	9.6E-3	9.9E0
UP_KEYWORDS	Fatty acid metabolism	4	1.4E-2	1.6E1
**Cluster 6**	**Enrichment score: 2.56**			
GOTERM_CC_DIRECT	Mitochondrial inner membrane	9	8.2E-4	9.8E-1
UP_SEQ_FEATURE	Transit peptide:mitochondrion	9	8.5E-4	1.2E0
UP_KEYWORDS	Transit peptide	9	1.4E-3	1.7E0
UP_KEYWORDS	Mitochondrion inner membrane	6	4.7E-3	5.7E0
GOTERM_CC_DIRECT	Mitochondrion	14	6.3E-3	7.2E0
UP_KEYWORDS	Mitochondrion	11	1.4E-2	1.6E1
**Cluster 7**	**Enrichment score: 2.54**			
GOTERM_BP_DIRECT	Protein folding	7	1.8E-4	2.5E-1
UP_KEYWORDS	Chaperone	5	8.9E-3	1.0E1
GOTERM_MF_DIRECT	Unfolded protein binding	4	1.5E-2	1.8E1
**Cluster 8**	**Enrichment score: 2.38**			
GOTERM_CC_DIRECT	Endoplasmic reticulum-Golgi intermediate compartment	6	1.4E-5	1.6E-2
GOTERM_BP_DIRECT	Retrograde vesicle-mediated transport, Golgi to ER	6	3.7E-5	5.2E-2
UP_KEYWORDS	ER-Golgi transport	6	3.9E-5	4.8E-2
GOTERM_CC_DIRECT	ER to Golgi transport vesicle membrane	4	1.7E-3	2.0E0
GOTERM_BP_DIRECT	COPII vesicle coating	4	2.8E-3	3.9E0
UP_KEYWORDS	Protein transport	9	3.2E-3	3.8E0
GOTERM_CC_DIRECT	Golgi membrane	9	5.1E-3	5.9E0
GOTERM_BP_DIRECT	ER to Golgi vesicle-mediated transport	5	6.3E-3	8.6E0
GOTERM_CC_DIRECT	Transport vesicle	4	9.0E-3	1.0E1
GOTERM_CC_DIRECT	Golgi apparatus	10	1.5E-2	1.7E1
GOTERM_CC_DIRECT	Endoplasmic reticulum-Golgi intermediate compartment membrane	3	3.4E-2	3.4E1
UP_KEYWORDS	Golgi apparatus	8	4.5E-2	4.4E1
GOTERM_BP_DIRECT	Transport	5	7.6E-2	6.7E1
GOTERM_BP_DIRECT	Intracellular protein transport	4	9.4E-2	7.6E1
UP_KEYWORDS	Cytoplasmic vesicle	5	1.4E-1	8.3E1
**Cluster 9**	**Enrichment score: 2.32**			
UP_KEYWORDS	Lipid metabolism	9	3.6E-4	4.4E-1
UP_KEYWORDS	NADP	5	6.8E-3	8.1E0
UP_KEYWORDS	Steroid biosynthesis	3	8.8E-3	1.0E1
UP_KEYWORDS	Lipid biosynthesis	4	2.5E-2	2.7E1
**Cluster 10**	**Enrichment score: 2.11**			
GOTERM_MF_DIRECT	Dolichyl-diphosphooligosaccharide-protein glycotransferase activity	3	7.8E-4	9.8E-1
GOTERM_CC_DIRECT	Oligosaccharyltransferase complex	3	8.8E-4	1.0E0
GOTERM_BP_DIRECT	Protein N-linked glycosylation via asparagine	3	1.4E-2	1.9E1
KEGG_PATHWAY	*N*-Glycan biosynthesis	3	4.0E-2	3.6E1
UP_KEYWORDS	Glycosyltransferase	4	7.0E-2	5.9E1

*After analyzing 84 CB2 specific interacting proteins (including CB2) with DAVID (https://david.ncifcrf.gov/home.jsp) 20 clusters were identified of which 10 were represented in this table with an enrichment score >2.*

Furthermore, we identified six proteins that were only expressed in the stable Strep-HA-CB2-HEK293 cells; the delta(24)-sterol reductase (DHC24), dehydrogenase/reductase SDR family member 7 (DHRS7), glutathione hydrolase 7 (GGT7), E3 ubiquitin-protein ligase HECTD3 (HECD3), an uncharacterized protein KIAA2013 (K2013), and the phospholipid scramblase 1 (PLS1).

### P62 Is an Interaction Partner of Cannabinoid Receptor CB2

**FIGURE 3:**
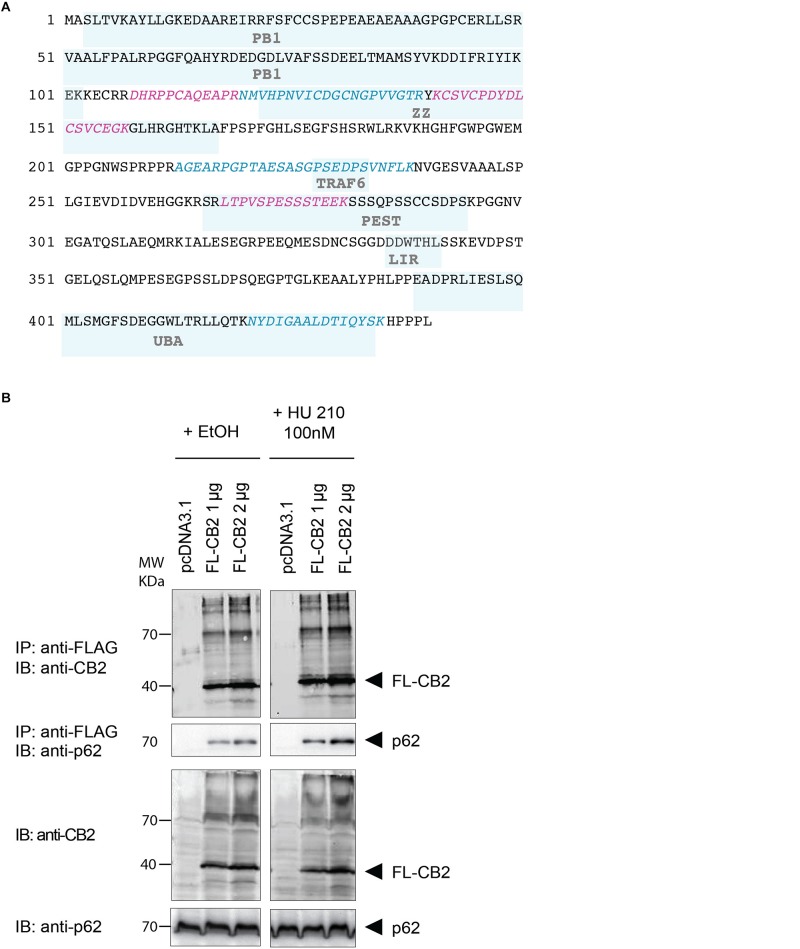
**(A)** Protein sequence of p62 with protein domains underlaid in light blue boxes (Phox and Bem1p domain (PB1), Zinc finger-type (ZZ), TNF receptor-associated factor 6 (TRAF6), LC3-interacting region (LIR), protein sequence rich in proline (P), glutamic acid (E), serine (S), and threonine (T) (PEST), UBA. The six peptides identified after tryptic digestion by mass spectrometry are highlighted in red and blue. **(B)** Verification of the interaction of CB2 receptors with p62 by co-immunoprecipitation (Co-IP) of transiently overexpressed flag tagged CB2 receptors (FL-CB2) with endogenously expressed p62 in HEK293 cells. The precipitated p62 protein intensity directly correlated with the amount of transfected CB2-receptor plasmid (either 1 or 2 μg) that was not influenced by 10 min stimulation with the CB2 agonist HU-210. Representative result of *n* > 3.

To verify the experimental approach and the obtained interactome list, we selected one specific protein with a high probability of interaction. To increase the likelihood of a direct protein interaction with CB2, this candidate should have a relatively low number of edges in our STRING network (Figure 2). Furthermore, the candidate should represent a signaling hub protein that might be involved in receptor and signaling crosstalk. Therefore, we chose p62/SQSTM1 for further experiments. The scaffold protein p62 is included in the 34 proteins of the functional annotation cluster 1 representing ER association. It is involved in TNF receptor signaling (Kim and Ozato, 2009) and in the development of PDB (Laurin et al., 2002) and functions as a signaling hub and as an autophagy adaptor (Katsuragi et al., 2015). The identification of p62 based on six specific tryptic peptides (i.e., AGEARPGPTAESASGPSEDPSVNFLK, CSVCPDYDLCSVCEGK, DHRPPCAQEA-PR, LTPVSPESSST EEK, NMVHPNVICDGCNGPVVGTR, NYDIGAALDTIQYSK) that represent 23.4% of the whole protein sequence (Figure 3A). Indeed, the quantification ratio of CB2 stable expressing versus control cells showed that p62 specific peptides were identified 52 times more in CB2-cells than in controls (Table 2).

In the next step, we verified the physical interaction of CB2 receptors with p62 by co-immunoprecipitation (Figure 3B). To this end we generated a Flag-tagged CB2 receptor using the expression vector pcDNA3.1 and transiently transfected HEK293 cells. We precipitated CB2 using beads carrying FLAG M2 antibodies and detected endogenous p62 by using an antibody against the human p62 protein (Figure 3B). The endogenous p62 was co-precipitated in Flag-tagged CB2-transfected (FL-CB2) cell extracts in an intensity directly correlated with the amount of transfected CB2-receptor plasmid (either 1 or 2 μg). In all pre-IP extracts (input) p62 was detectable in comparable intensities whereas in control transfected protein extracts (pcDNA3.1) no p62 band was visible after immunoprecipitation. The stimulation with a CB2 receptor agonist (HU-210) did not alter the interaction (Figure 3B). These results strongly indicate that CB2 receptor interacts with p62 and that stimulation of CB2 does not interfere with this interaction.

### CB2 Receptors Colocalize With p62 Vesicles Mainly in Plasma Membrane Associated Regions

**FIGURE 4:**
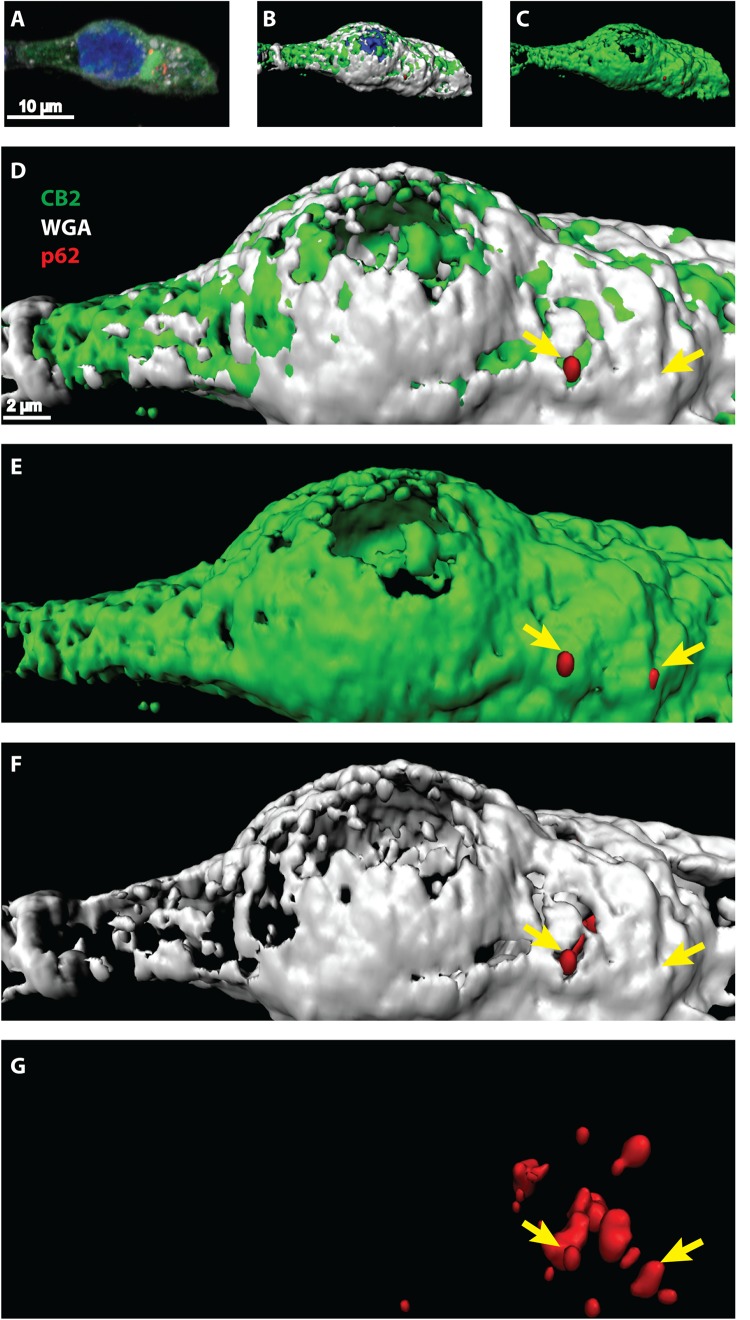
**(A–G)** Confocal light microscopy of HEK293 cells transiently expressing CB2 receptors. **(A)** Immunohistochemical staining of CB2 (green), p62 (red), and DAPI (blue). The membrane was stained by Texas-red conjugated wheat-germ-agglutinin (white) and DAPI revealed the localization of the nucleus. **(B–G)** 3D reconstruction modeling of confocal images using a segmentation algorithm to distinguish single signal from different channels. **(D–G)** Higher zoom in of 3D reconstructed cell showing a strong overlap of CB2 positive areas with the membrane marker (white). P62 was found in vesicular like structures that intermingled with membranal CB2 positive areas (yellow arrows).

Once activated, GPCRs are typically internalized and either degraded or recycled to the membrane for another activation cycle. Degradation can occur via lysosome and autophagy pathways where the scaffolding protein p62 is involved in. Since we showed that CB2 interacts with p62 in whole cell lysates we wondered at which cellular compartment this interaction is taking place. We performed immunocytochemical studies of HEK293 cells expressing endogenously p62 and transiently CB2. For surface membrane staining we used WGA-texas red labeled probes. Confocal microscopy revealed a broad and predominant membrane localization of transiently expressed CB2 receptors (Figure 4 and Supplementary Figure 2) but a localization of p62 in dotted and vesicular like structures that are most probable lysosome and autophagosome areas. Because of the relatively strong heterologous expression of CB2 we selected the p62 positive areas and analyzed the co-localization with CB2 receptors using the Manders coefficient and obtained a mean value of 0.46 ± 0.07. Additionally, we aimed to better visualize and to clearly distinguish localizations of both proteins in co-localization with the membrane marker. Therefore, we performed a 3D reconstruction modeling of confocal images. With this approach we remodeled the p62 expression in red vesicular structures (Figures 4D–G). We used for our analysis a segmentation algorithm that eliminates signals below a threshold to visualize the p62 vesicles optimally. Thus, any signals of p62 that are weaker are not present in the 3D-reconstruction images. In white/gray we visualized the areas that were positively stained with membrane marker (Figures 4D,F) that intermingled with green CB2 receptor signals (Figures 4D,E). Our 3D reconstruction images indicated that those p62 vesicles were surrounded by CB2 positive areas that were adjacent to the surface membrane (Figure 4D). These results demonstrate that about half of the p62 positive areas co-expressed CB2 and that these p62-CB2 vesicles are at the cell membrane.

### Intracellular Regions of CB2 Receptor Are Not Responsible for the Interaction With p62

**FIGURE 5:**
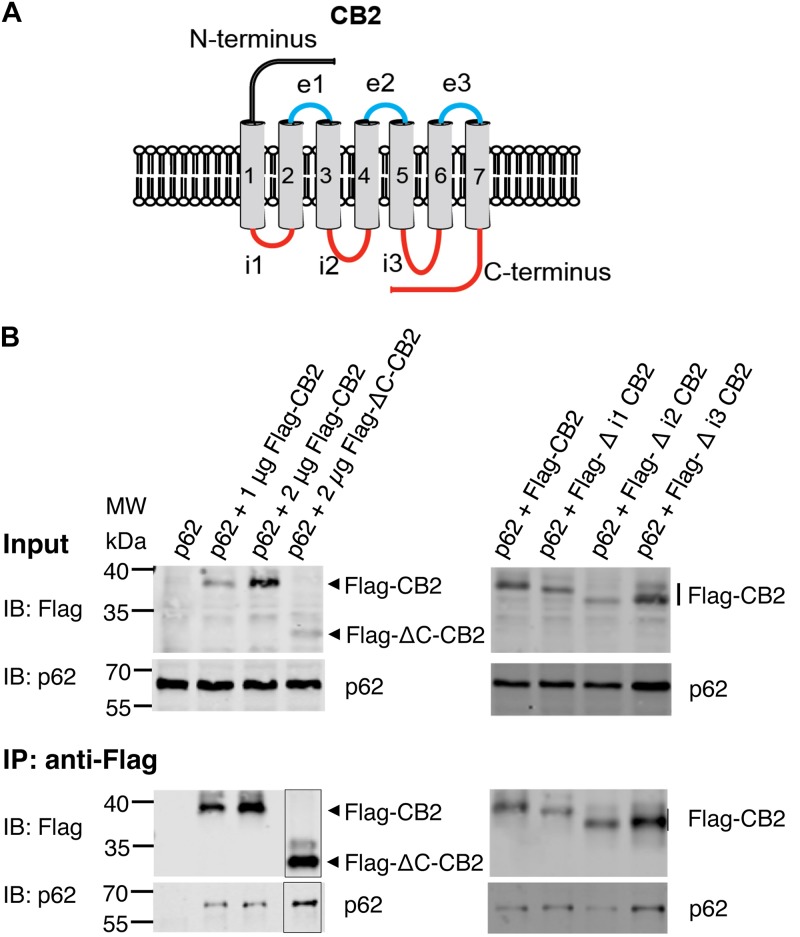
**(A)** Schematic drawing of CB2 receptor protein with extracellular N-terminus and three extracellular loops (e1, e2, e3), seven transmembrane domains, and three intracellularly localized loops (i1, i2, i3) and the C-terminus. The red marked intracellular regions were deleted in CB2 expression plasmids and used for following Co-IP studies. **(B)** Representative Western blot results of CB2 precipitations using Flag antibody with lysates of HEK293 cells transiently transfected with p62-WT (1 μg) and Flag-CB2 constructs with the respective deletions (ΔC-CB2, Δ i1-CB2, Δ i2-CB2, Δ i3-CB2). Considering lower expression levels of deletion constructs compared to WT-full length Flag-CB2, the co-precipitated p62 protein levels were similar in all approaches. The IP figure was grouped from cropped parts of the same gels/blots indicated by the border line. This experiment has been performed at least two times independently.

Since p62 is localized intracellularly we wondered whether p62 interacts with intracellular domains of CB2. We designed expression constructs deleting the three intracellular loops and the C-terminus of the CB2 receptor (Figure 5A). Surprisingly none of these deletion constructs altered the interaction of CB2 with p62 in co-IP studies if correlated to the different expression levels in the input and the IPs (Figure 5B). We conclude from this experiment that it is not individual intracellular loops that are responsible for the interaction, but rather that multiple domains are involved in the interaction with p62. Because receptor structure is strongly dependent on the transmembrane domains, we have not investigated further deletion constructs of CB2 in this assay.

### ZZ Domain of p62 Is Important for Interaction With CB2

**FIGURE 6:**
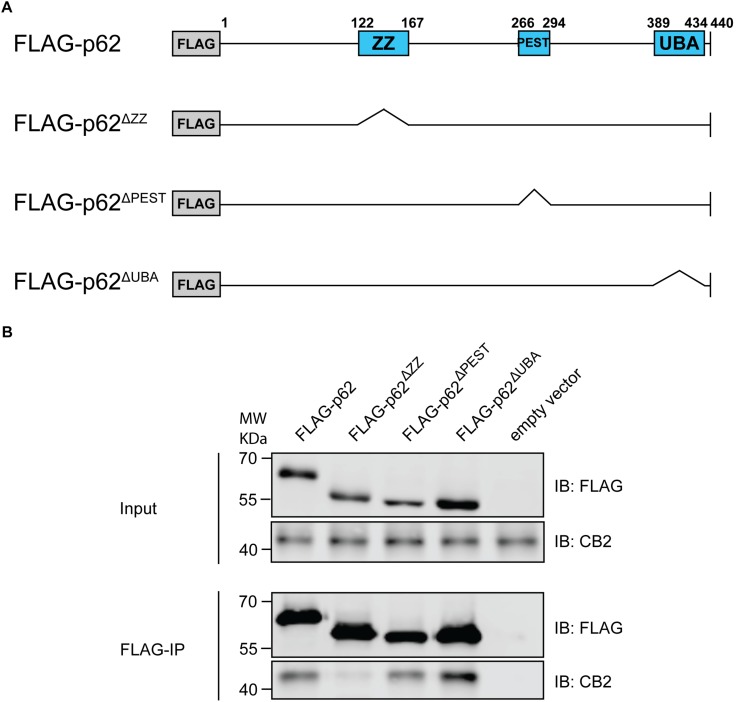
**(A)** Schematic illustration of Flag-p62 deletion constructs for ZZ, PEST, and UBA motifs that were used for following co-immunoprecipitation experiments to identify interaction region. **(B)** Stably expressing HA-CB2 HEK293 cells were transiently transfected with different Flag-p62-deletion constructs, respectively. Protein extracts were used for precipitation of Flag-p62 using Flag antibody. The expression levels in input samples for CB2 and p62 were comparable in all constructs. Immunoprecipitation result of Flag-p62 ΔZZ revealed a significantly reduced coprecipitation of CB2 indicating that this domain in p62 is involved in the interaction with CB2. Representative results of three independent experiments were presented.

P62 is a cargo and multifunctional protein carrying several motifs and domains responsible for the interaction with a variety of proteins (Moscat et al., 2007). Therefore, we aimed to identify the motif responsible for CB2 interaction and co-expressed CB2 with deletion constructs of p62 lacking the PB1 domain and the binding motifs ZZ and UBA respectively (Figure 6A). Only the ZZ deletion construct failed to co-precipitate CB2 indicating that this region is important for the interaction of p62 with CB2 (Figure 6B). All other investigated p62 constructs including the UBA-domain deletion protein resulted in CB2 receptor co-precipitations that were comparable to positive controls. We therefore showed that CB2 interacts with p62 via the ZZ-domain.

In summary, we identified the CB2 interactome in HEK293 cells consisting of 83 interacting proteins involved in, e.g., biosynthesis, trafficking, and signaling. The identified proteins are mainly localized at the ER and other membrane structures in the cell and in intracellular compartments. The autophagy receptor p62 was identified by six unique peptides as an interactor with a very high probability of interaction with CB2 receptor. In our investigations we focused on p62-CB2 complex formation and could verify that CB2 receptor interacts with the ZZ domain of p62 at membranous structures close to the cell surface. Future investigations will concentrate on the characterization of the interaction and its physiological impact in the cell.

## Discussion

Investigations to identify GPCR interactomes are valuable approaches for the understanding of receptor function and methods like AP-MS and MYTH enabled first reproducible identifications of GPCR interactomes (Kittanakom et al., 2014; Sokolina et al., 2017). In the present work we applied tandem mass spectrometry and identified a CB2 receptor interactome consisting of 83 proteins and characterized its interaction with p62 in more detail. The identified interactome especially contains protein networks involved in signaling, biosynthesis and trafficking like calnexin, tubulin chains, and TMED10.

One major concern in the investigations of protein interactomes is the reduction of false-positive findings, which we minimized by the inclusion of only those proteins that were identified by at least two unique peptides. To further drastically reduce the false discovery rate a method established by Glatter et al. (2009) was applied and analyzed the CB2 receptor interactome in triplicates with a reproducibility of at least 90%. Thus, the identified interaction data in our study should represent data with a high level of robustness and reliability. The lack of specific CB2 antibodies (Cecyre et al., 2014) limited our experimental set up to cultured model cells reconstituted with an epitope- tagged CB2 receptor. But the Flp-mediated recombination enabled an insertion of CB2 receptor cDNA into a single genomic locus, which minimize the overexpression levels in this model system. Nevertheless, the relatively high biosynthesis of the CB2 protein could be represented in the interaction network, since GO annotation analysis showed that a relatively high portion of the CB2 interacting proteins were associated with ER and with proteins involved in biosynthesis and degradation pathways. Sec61A1, calnexin, and DNAJA2 are examples for interactors that are functionally relevant in translocation of newly synthesized polypeptides to the ER, correct folding and further trafficking of proteins. Previously, binding of calnexin to the GPCRs rhodopsin and delta-opioid receptor and an interaction of the chaperone of the J protein family DNAJ2B to rhodopsin had been demonstrated (Chapple and Cheetham, 2003; Rosenbaum et al., 2006; Tuusa et al., 2010) emphasizing the relevance of our investigations.

The identified GTP binding proteins are involved in a variety of functions ranging from recycling and trafficking to microtubule stability, cytokinesis, and mitochondrial biosynthesis. The signaling alpha subunits of guanine nucleotide-binding proteins (GNA proteins) were also identified in the list of interacting proteins. Interestingly, GNAS1 and the GNAI3 proteins were present by a single unique peptide but not GNAI1 or GNAI2. The CB2 receptor has mainly been associated with G alpha-i subunit coupled responses and signaling via G alpha s coupled subunits has, to our knowledge, not been reported for CB2 signaling. Possibly, our results hint to similar options of CB2 receptor signaling via GNAS and GNAI3 as has already been described for CB1 receptor (Mukhopadhyay et al., 2000; Finlay et al., 2017).

Findings for other GPCRs further support our newly identified interactions of CB2 receptors. For example, a direct interaction of the C-terminus of alpha2B-adrenergic receptor (alpha2B-AR) with tubulin has been demonstrated to mediate receptor trafficking from ER to cell surface (Duvernay et al., 2011). Our findings of an interaction of CB2 with the tubulin chains, TUBB2B and TUBA1C, might implicate a similar role for proper receptor transport. In line with this, the interactions with the trafficking proteins, TMED2 and TMED10, might hint at an endocytic transport of resensitized CB2 receptor via these proteins as has already been demonstrated for opioid receptor OPRM1 and purinergic nucleotide receptor P2RY4 (Luo et al., 2011). Our results open the path for future studies to investigate, e.g., if CB2 receptor trafficking and homeostasis is coordinated by its newly identified interacting proteins.

Those six proteins that were only identified in CB2-expressing cells DHC24, DHRS7, GGT7, HECD3, KIAA2013, and PLS1 are not forming an interaction network. Nevertheless, the interaction of CB2 with DHC24, a sterol intermediate catalyzing enzyme, and PLS1, an enzyme that flips phosphatidylserines between membrane leaflets, strongly argues for a functional importance in metabolism of lipid molecules connected to the cell membrane that might be of importance, e.g., for viral entry (Williams et al., 2014; Cheshenko et al., 2018).

The reliability of our data is supported by the fact that we have analyzed one protein by Co-IP and could verify the results obtained by the AP-MS approach. For the detailed interaction analysis, we focused on the scaffold protein p62. Our results show a localization of CB2 at p62 positive vesicles that are also WGA positive. WGA selectively binds to *N*-acetylglucosamine and *N*-acetylneuraminic acid (sialic acid) residues at the cell membrane (Wright, 1984). Because of the threshold that we used for 3D-reconstructions, the amount of p62 proteins cannot be compared between the models of confocal images and Western blots.

The protein p62 acts as a cargo protein in autophagy and proteasome targeting by its UBA and PB1 domain (Cohen-Kaplan et al., 2016) and regulates the activation of NF-κB (Sanz et al., 1999). We identified the ZZ domain as a main contributor to the interaction with CB2. Recently, binding of the ZZ domain of p62 to arginylated BiP (the ER-localized homolog of Hsp70) was demonstrated to be involved in p62 oligomerization and interaction with the autophagy marker LC3 leading to p62 targeting to autophagosomes and selective lysosomal co-degradation of R-BiP and p62 together with associated cargoes (Cha-Molstad et al., 2015). The enzyme responsible for the N-terminal arginylation is catalyzed by arginyltransferase 1 (ATE1)-encoded Arg-transfer RNA transferases (Cha-Molstad et al., 2015), which was also identified in the CB2 interactome. Therefore, the ZZ domain is particularly important for redirecting N-end rule substrates to the autophagy pathway (Kwon et al., 2018). Thus, biologically the p62-CB2-interaction could be of relevance in cells for an autophagosomal degradation and clearance of CB2 receptors. However, the interaction via the ZZ-type zinc finger motif of p62 also hints at a signaling complex formation as, e.g., the binding to RIP1 protein that links PKCz to form a signaling complex (Festjens et al., 2007; Lin et al., 2013). RIP1 kinase binds to TNF receptor influencing NF-κB and p38 MAPK signaling pathways (Lin et al., 2013), which are also activated by CB2 receptor expression or activation (Dhopeshwarkar and Mackie, 2014; Toguri et al., 2014). Generally, CB2 receptor functions are connected to co-stimulatory effects of, e.g., cytokines, which are then modulated by the GPCR action (Cabral et al., 2015a). Because dysfunction of p62 as well as CB2 receptors are involved in neurodegenerative diseases, inflammation, cancer and bone biology (Pacher and Mechoulam, 2011; Duran et al., 2016; Sanchez-Martin and Komatsu, 2018), it has to be clarified in future experiments if the complex formation of p62 and CB2 is involved in these pathophysiologic functions. Since p62 acts as a signaling platform for, e.g., TNF receptor 1, IL-1 receptor, and RANK receptor (Moscat et al., 2007) and is involved in Toll-like receptor 4 (TLR4) – mediated autophagy (Fujita et al., 2011), our findings might indicate that this platform could function as a signaling hub, which enables the crosstalk of different receptors with CB2 protein. Recently identified interaction between CB2 and TLR4 and their co-expression, co-precipitation and co-functioning in macrophage activation and cancer progression (Xiang et al., 2018) might be one possible example for these protein complexes.

In general, the CB2 receptor is emerging as an attractive therapeutic target for immunomodulation (Cabral et al., 2015b). The potential of CB2 antagonists as a target for treating cancer (Xiang et al., 2018) and liver fibrosis (Mallat et al., 2013) and of CB2 receptor agonists in the treatment of neuropathic pain, neuroinflammation, and neurodegenerative disorders (Guindon and Hohmann, 2008; Lunn et al., 2008; Racz et al., 2008a, b; Contino et al., 2017) hint to the need of molecular insights into the activation mechanism of CB2.

Recently, the crystal structure of the human CB1 and CB2 receptors have been revealed (Hua et al., 2016; Krishna Kumar et al., 2019; Li et al., 2019) and led to the discovery of an opposing functional profile of CB2 antagonism versus CB1 agonism (Li et al., 2019). The clarification of ligand selectivity or function will provide new insights into precise modulation of the ECS by structural analysis, molecular docking and mutagenesis studies (Li et al., 2019). A combination of crystal structure data with results from protein interactome screenings will contribute to facilitate rational drug design and understand mode of action of agonists or antagonists/inverse agonists. Our data add a first step toward this aim, but in future it would be of additional relevance to investigate the CB2 interactome by applying a broader spectrum of cannabinoids.

Despite the use of a heterologous expression system in HEK293 cells, the present work reveals for the first time a CB2 interactome and represent a basal and representative overview. HEK293 cells have been shown to express a variety of GPCR class A transcripts like histamine, dopamine, chemokine, and adenosine receptors (Atwood et al., 2011). Thus, the machinery for GPCR biosynthesis, signaling, and trafficking is present in these cells and is available for the over-expressed cannabinoid receptors that are functional in HEK293 cells (Nagler et al., 2016; Hunter et al., 2017). Nevertheless, investigations of native CB2 expressing cells like lymphocytes and macrophages might result into partially different interactomes. It will therefore be valuable to perform similar screening approaches in different cell types under stimulation with different ligands and systematically compare the interactomes to get a specific and detailed understanding of the CB2 receptor function.

In summary, with the identification of new protein interaction partners for CB2, our findings provide new molecular insights contributing for a better understanding of CB2 receptor biosynthesis, signaling and functions that might account to improve therapeutic strategies of this clinically relevant protein.

## Data Availability

The datasets generated for this study can be found in the Supplementary Data to this manuscript.

## Author Contributions

MK was responsible for research design. AS, LM, SR, CK, MS, LP, BM, and MK were involved in the generation of expression plasmids, IP experiments, the analysis of these experiments, and figure preparation. ND performed the IPs with deletion constructs of CB2 receptor loops and prepared the figures. AF, SR, and MK conducted the microscopic experiments including analysis, figure preparation, and interpretation. MK performed the bioinformatic analyzes that were approved by HK. MK, AS, and MR wrote or contributed to the writing of the manuscript. All authors read and approved the final manuscript.

## Conflict of Interest Statement

The authors declare that the research was conducted in the absence of any commercial or financial relationships that could be construed as a potential conflict of interest.

## Footnotes

1 https://david.ncifcrf.gov/

## Abbreviations

AOBS: Acusto Optic Beam Splitter
AP-MS: affinity purification coupled to mass spectrometry
BP: biological process
CB1: cannabinoid receptor 1
CB2: cannabinoid receptor 2
CC: cellular component
DDM: dodecylmaltosid
DMEM: Dulbecco’s modified Eagle’s medium
FBS: fetal bovine serum
FCS: fetal calf serum
GPCRs: G-protein coupled receptors
HyD: high sensitive hybrid detectors
LPS: lipopolysaccharide
MF: molecular function
MYTH: modified membrane yeast to hybrid
NF- κ B: nuclear factor- light-chain-enhancer of activated B cells
PDB: Paget’s disease of bone
RANK: receptor activator of nuclear factor-kappa B
RANKL: receptor activator of nuclear factor-kappa B ligand
RT: room temperature
TLRs: toll like receptors
TNF: tumor necrosis factor
TRAF6: TNF-receptor associated factor 6
TUBA1C: tubulin alpha-1C
TUBB2B: tubulin beta-2B
UBA: ubiquitin-associated domain
WGA: wheat germ agglutinin.
