# Supplementary material for: Systematic Affinity Purification Coupled to Mass Spectrometry Identified p62 as Part of the Cannabinoid Receptor CB2 Interactome
Source: Front Mol Neurosci. 2019 Sep 20;12:224. doi: 10.3389/fnmol.2019.00224 (PMC6763791; doi:10.3389/fnmol.2019.00224)
Supplement: Supplementary file 2 [file Image_1.pdf]

Supplementary Figure 1

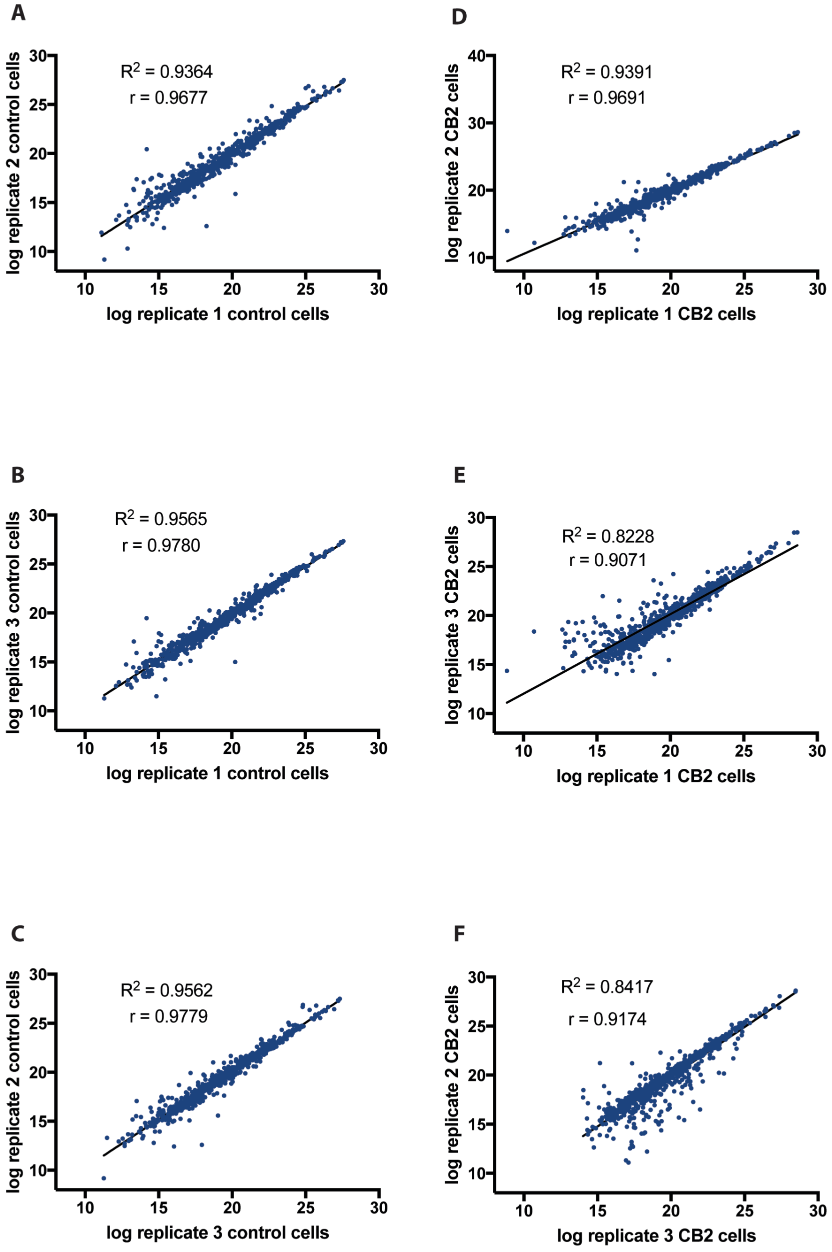

Supplementary Figure 1.
Analysis of Strep-HA-CB2 AP-MS replicates variation of untransfected HEK293 cells (A to C) and Strep-HA CB2 expressing HEK293 cells (D to F). Plotted are the intensities of the proteins identified in all three replicates. Pearson correlation coefficient is r and its square is given as R^2.

Supplementary Figure 2

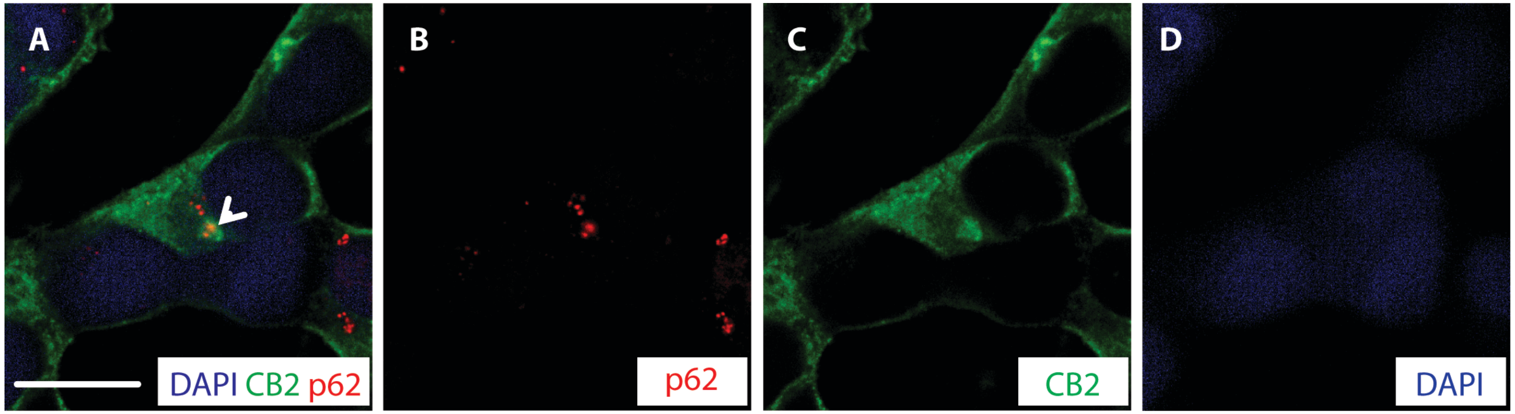

Supplementary Figure 2.
Confocal single plane light microscopy of HEK293 cells transiently expressing CB2 receptors.
A) Overlay image of immunohistochemical staining of p62 (red, B), CB2 (green, C), and DAPI (blue, D). Endogenous p62 was found in vesicular like structures that intermingled with CB2 positive areas (white arrow head). Scale bar = 10µm.
